# Supplementary material for: Extinction vortices are driven more by a shortage of beneficial mutations than by deleterious mutation accumulation
Source: bioRxiv. 2026 Mar 10:2024.10.25.620329. Preprint. [Version 4] doi: 10.1101/2024.10.25.620329 (PMC13014150; doi:10.1101/2024.10.25.620329)
Supplement: Supplement 1 [file media-1.pdf]

Supplementary Information

Extinction vortices are driven more by a shortage of beneficial mutations than by deleterious mutation accumulation

Walid Mawass[1,2], Joseph Matheson[1,3], Ulises Hernández[1], Jeremy J. Berg[2], Joanna Masel[1]

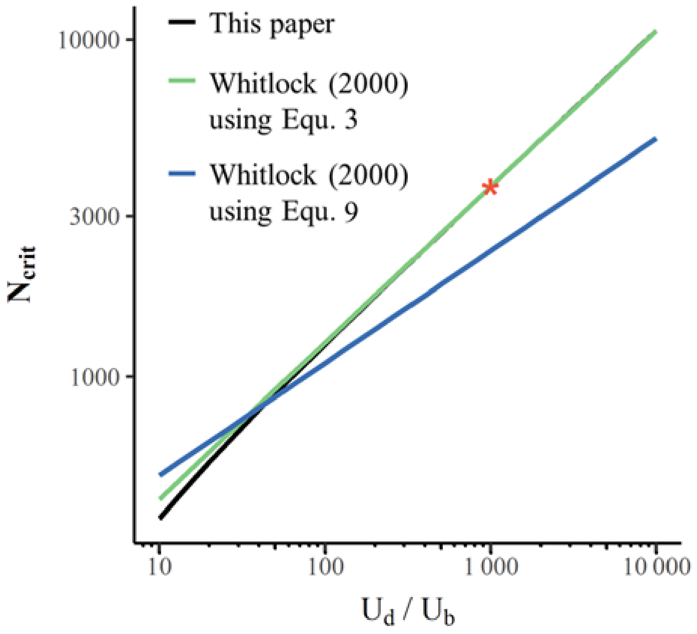

Figure S1. Our analytical treatment of the critical population size is better approximated by Whitlock's (2000) Equation (3) than by his Equation (9). To use Equation (3), we take v_d from there rather than from the approximate Equation (4) to produce an alternative to Equation (9). Whitlock's predictions were multiplied by 2 to account for the higher genetic drift of the Moran process relative to the Wright-Fisher model. Results are shown for $U_d = 2$ and varying U_b, but we confirmed that results varying U_d instead, while holding U_b =0.002, are superimposable. $\bar{s}_d$ = -0.009 via the DFE from Kim et al. (2017) from their Table 2 (row 9) and $\bar{s}_b$ = 0.001. Red star represents our example of U_d/U_b=1000 in main figures 2 and 6 with no environmental change.

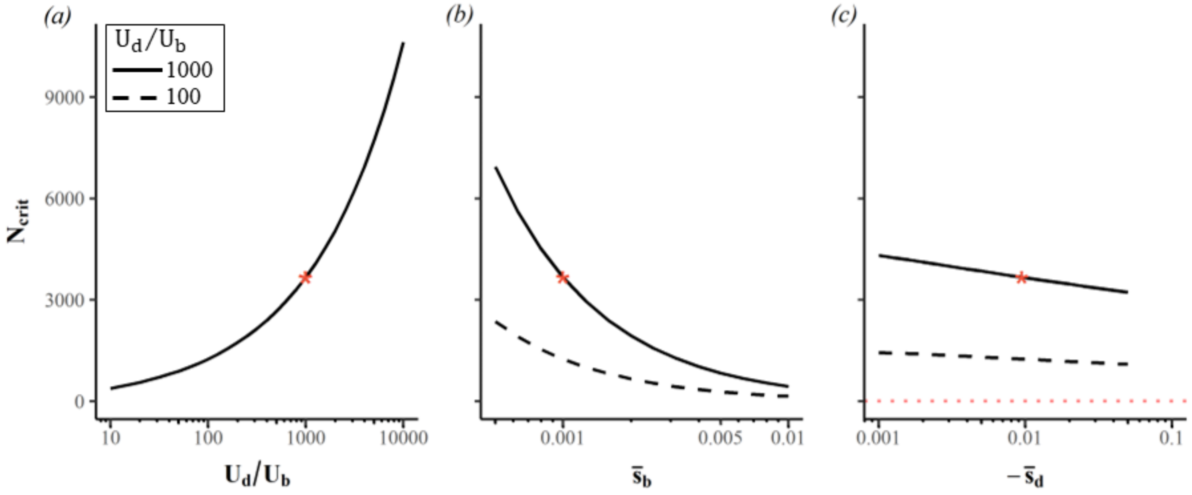

Figure S2. $\boldsymbol{N_{crit}}$ **dependence on parameter value choices.** Linkage equilibrium is assumed, with no environmental change. (a) N_{crit} is much lower when beneficial mutations are abundant (low U_d/U_b). N_{crit} drops less with (b) stronger beneficial effects and less again with (c) stronger deleterious effects. Panels (a) and (b) use $\bar{s}_d$ = 0.009 via the DFE from Kim et al. (2017). Panels (a) and (c) use $\bar{s}_b$ = 0.001. U_d = 2 throughout, but results for different U_d are superimposable. The red star indicates the example shown in Figure 2 with no environmental change.

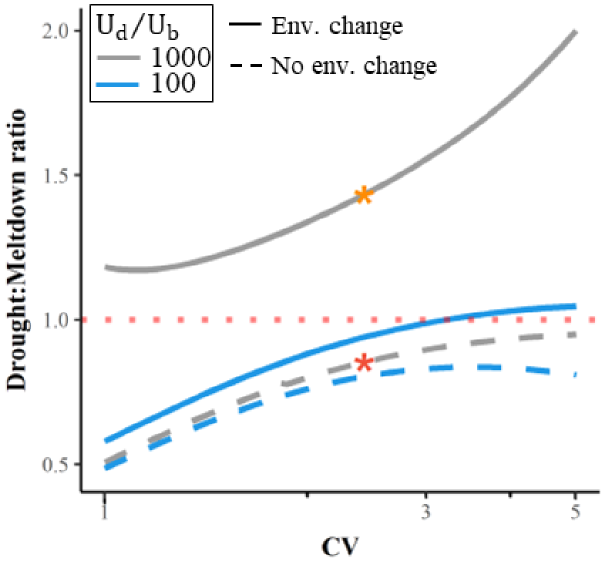

Figure S3. **Drought becomes more important relative to meltdown when the deleterious DFE is overdispersed.** Linkage equilibrium is assumed. To isolate the effect of the coefficient of variance (CV), we hold the mean deleterious effect size constant at the $\bar{s}_d$ corresponding to the DFE from Kim et al. (2017), and adjust the parameters of the gamma distribution. Beneficial mutations have an exponential distribution with $\bar{s}_b$ = 0.001. The red and orange stars indicate the two scenarios in Figure 2, with no environmental change and with environmental change (δ_{env}= -1.5×10^{-5} per generation), respectively.

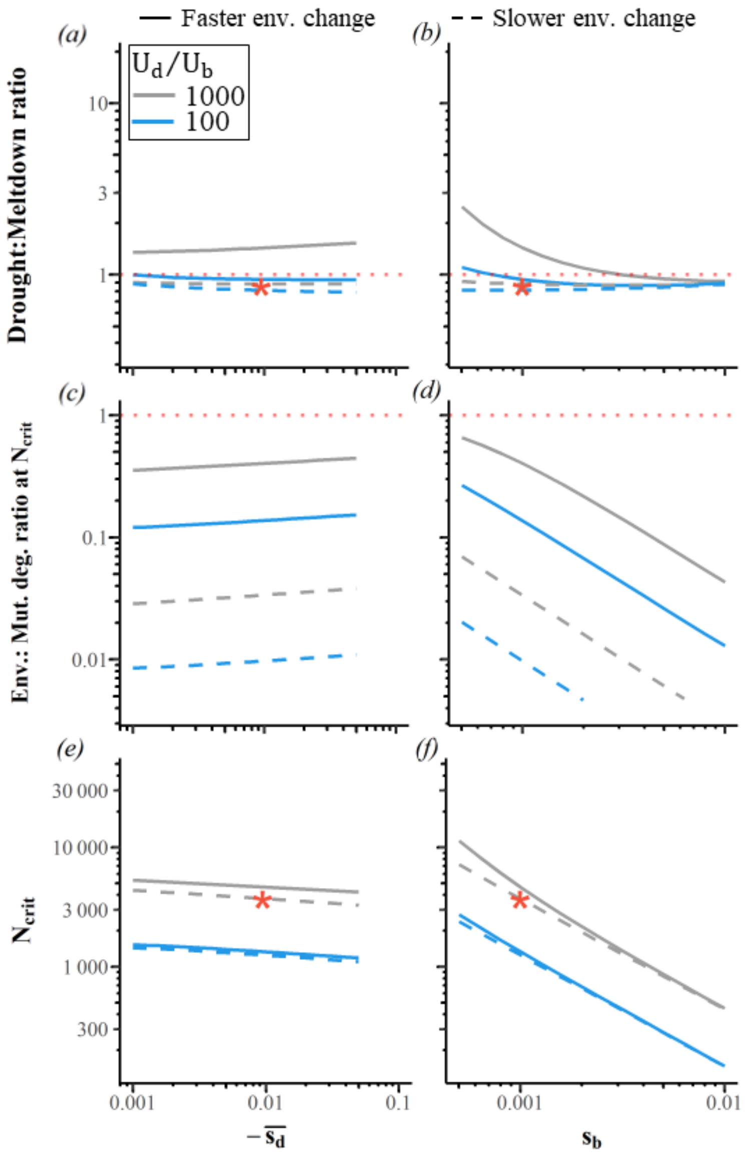

Figure S4. The drought : meltdown ratio is largely insensitive to selection coefficients, but rises for small $\bar{s}_b$. As in Figure 5, the red star indicates the example shown in Figure 2 with no environmental change. "Faster env. change" (blue lines) represents a fitness reduction of δ_{env}= -1.5×10^{-5} per generation, and "slower env. change" (black lines) represents δ_{env} = -10^{-6}. Results are shown for $U_d = 2$ and varying U_b, but results varying U_d instead, while holding U_b =0.002, are qualitatively similar. The y-axes scales match those in Figure 5, to facilitate comparison.

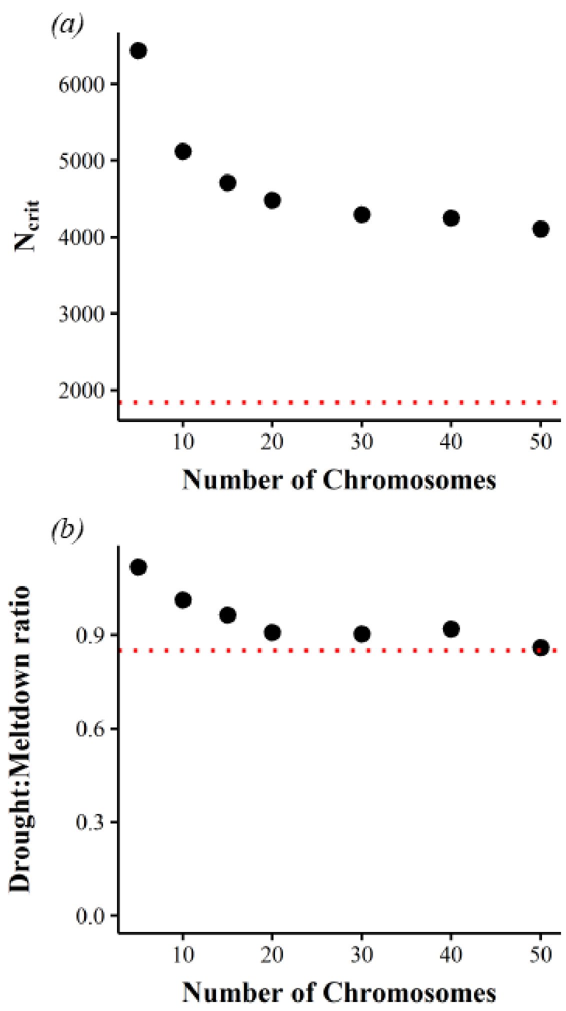

Figure S5. **More recombination does not make N_{crit} converge to the expectation for independent loci (a), but this does not affect the drought : meltdown ratio (b).** $\bar{s}_b$ = 0.001, U_b = 0.002, U_d = 2, $\bar{s}_d$ = -0.009 via the DFE from Kim et al. (2017), L = 100 per chromosome. Red dashed lines show analytical expectations in the absence of LD.

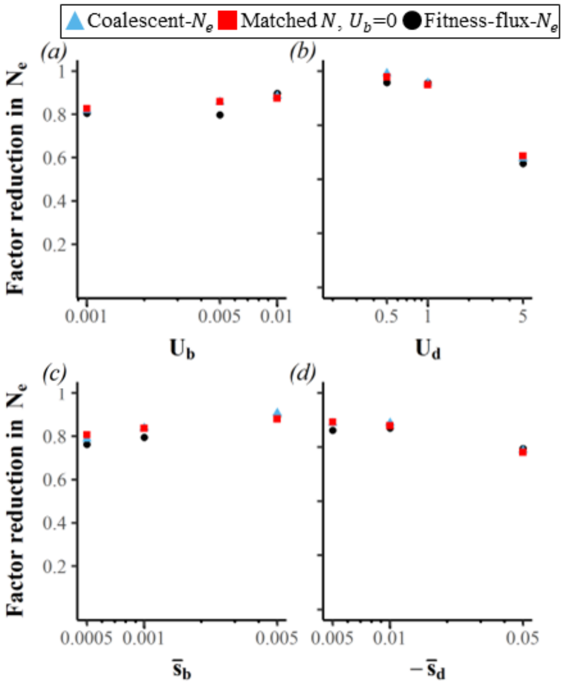

Figure S6. **The reduction in effective population size below census size is driven by background selection.** The reduction derived from fitness fluxes $\frac{\text{analytical } N_{crit}}{\text{simulated } N_{crit}}$ (black circles) is close to N_e/N from coalescence time (blue triangles). Both align with coalescent N_e/N derived from simulations of matched census N with deleterious mutations alone (U_b = 0, red squares). Where not otherwise specified, $\bar{s}_b$ = 0.001, U_d = 2, U_b = 0.02, and $\bar{s}_d$ = -0.009 via the DFE from Kim et al. (2017). There is no environmental change.

References

Kim BY, Huber CD, Lohmueller KE. 2017. Inference of the Distribution of Selection Coefficients for New Nonsynonymous Mutations Using Large Samples. Genetics. 206(1):345–361. https://doi.org/10.1534/genetics.116.197145

Whitlock MC. 2000. Fixation of New Alleles and the Extinction of Small Populations: Drift Load, Beneficial Alleles, and Sexual Selection. Evolution. 54(6):1855–1861
